# Linking grandiose and vulnerable narcissism to managerial work performance, through the lens of core personality traits and social desirability

**DOI:** 10.1038/s41598-024-60202-7

**Published:** 2024-05-28

**Authors:** Anna M. Dåderman, Petri J. Kajonius

**Affiliations:** 1 Department of Social and Behavioral Studies, University West, Trollhättan, Sweden; 2 Department of Psychology, Lund University, Box 213, 221 00 Lund, Sweden

**Keywords:** Narcissism (SD3, HSNS), HEXACO (Mini-IPIP6), Individual work performance (IWPQ), Socially desirable responding (BIDR 6), Human behaviour, Risk factors

## Abstract

While grandiose narcissism is well-studied, vulnerable narcissism remains largely unexplored in the workplace context. Our study aimed to compare grandiose and vulnerable narcissism among managers and people from the general population. Within the managerial sample, our objective was to examine how these traits diverge concerning core personality traits and socially desirable responses. Furthermore, we endeavored to explore their associations with individual managerial performance, encompassing task performance, contextual performance, and counterproductive work behavior (CWB). Involving a pool of managerial participants (*N* = 344), we found that compared to the general population, managers exhibited higher levels of grandiose narcissism and lower levels of vulnerable narcissism. While both narcissistic variants had a minimal correlation (*r* = .02) with each other, they differentially predicted work performance. Notably, grandiose narcissism did not significantly predict any work performance dimension, whereas vulnerable narcissism, along with neuroticism, predicted higher CWB and lower task performance. Conscientiousness emerged as the strongest predictor of task performance. This study suggests that organizations might not benefit from managers with vulnerable narcissism. Understanding these distinct narcissistic variants offers insights into their impacts on managerial performance in work settings.

## Introduction

Our comprehension of the impact of vulnerable narcissism on leadership remains in its nascent stages, and this study aimed to establish a data-driven foundation for further understanding. This endeavor is crucial for empirically examining vulnerable narcissism within organizational contexts. Research on CEOs personality predominantly centers on grandiose narcissism^1–3^ or core personality traits such as conscientiousness and neuroticism^4^, but not on vulnerable narcissism. Recent work by Miller et al.^5^ underscores the current knowledge and gaps in our understanding of various narcissistic constructs, emphasizing the necessity for additional exploration of narcissistic vulnerability itself.

While extensive research exists on grandiose narcissism among managers, the focus on vulnerable narcissism in managerial roles remains scant. Our aim was to explore two variants of narcissism—grandiose and vulnerable—within managerial roles, comparing and contrasting these traits with those observed in the general population. Moreover, within the managerial sample, we aimed to investigate the divergent correlations between both forms of narcissism and core personality traits, alongside examining their correlations with socially desirable responding. Additionally, we aimed to explore the associations between both variants of narcissism and managerial performance, encompassing task performance, contextual performance, and counterproductive work behavior.

To our knowledge, comparative analyses between managers and the general population regarding the level of these two narcissistic variants are lacking. However, it is recognized that employees in senior leadership positions tend to exhibit elevated scores of narcissism, as evaluated both by self-ratings and ratings provided by their subordinates^6^. Are managers more prone to grandiose narcissism and less susceptible to vulnerability compared to the general population? Would we anticipate observing a comparable pattern of divergent correlations between these two narcissistic variants and core personality traits in a sample of managers as is typically observed in the general population?

The focal point of managerial research, applying intricate methodologies encompassing numerous scales, predominantly centers on task performance^7,8^. Conversely, less attention is allocated to exploring alternative facets of individual performance, such as contextual performance. Previous studies have primarily examined the grandiose variant of narcissism in relation to work performance, revealing that narcissistic managers often overestimate their performance^9^, which may be related to socially desirable responding. However, it remains unclear whether this overestimation extends to other form of performance, including contextual performance. It would be intriguing to explore whether vulnerable narcissism predicts counterproductive work behavior, potentially indicating a need to prioritize the recruitment of managers with lower levels of vulnerable narcissism.

### Grandiose and vulnerable narcissism defined

Morf et al.^10^ offered a comprehensive delineation of the background and historical context surrounding the construct of narcissism. They traced its origins back to early depictions by scholars such as Havelock Ellis and Freud, who characterized narcissistic individuals as those excessively preoccupied with self-investment and the preservation of their ego, often to the detriment of others. Morf et al.^10^ described narcissism as “self-enhancer personality” (p. 399). According to Morf and Rhodewalt^11^ persons with narcissism are full of paradoxes, characterized by self-aggrandizement and self-absorption, yet paradoxically susceptible to perceived threats and overly sensitive to feedback. They exhibit emotional volatility across a spectrum from euphoria to despair and rage, whether in the role of a friend, boss, or romantic partner. Despite their charm and social adeptness, they display a marked insensitivity towards the feelings, desires, and needs of others. Initial attraction to such personalities may be common, only to be overshadowed by exhaustion from their incessant cravings for admiration and attention.

Narcissism, when considered at more formal and heightened levels, is classified as a personality disorder according to the *Diagnostic and Statistical Manual of Mental Disorders*^12^ (DSM-5; APA). However, it is crucial to note that this study focuses on narcissism as a personality trait rather than as a diagnosable disorder.

For the last few decades, the Dark Triad traits—narcissism, Machiavellianism, and psychopathy, representing three socially aversive personality traits as outlined by Paulhus and Williams^13^—have garnered considerable attention as a focal point in research. This study focuses on narcissism, often regarded as comparatively less socially aversive in the workplace compared to other traits within the Dark Triad. Narcissism is a personality trait characterized by an inflated sense of self-importance and a need for excessive admiration^11^. There are two, at least in nonclinical samples unrelated, main variants of narcissism: grandiose and vulnerable (see review by Jauk and Kanske^14^). Grandiose narcissists exaggerate their abilities, are arrogant, and seek power, while vulnerable narcissists are insecure yet still feel important.

In addition to the entitled behavior often associated with the grandiose variant of narcissism, vulnerable narcissism manifests in hypersensitivity and anxiety^15,16^. It tends to correlate with deflated self-esteem, depression, anxiety, and a lack of concern for others’ needs. Unlike the grandiose variant, vulnerable narcissism is not linked theoretically or empirically to overt self-reporting, such as bragging about successful organizational behaviors in the past^17,18^. Displaying proactive behaviors at work stems from a drive for status and power within the organization, rather than the characteristics of vulnerable narcissism.

### The dichotomy of narcissistic grandiosity in organizational environments

In organizational settings, grandiose narcissism exhibits a dual nature, correlating with outcomes that can be either advantageous or detrimental. Employees with grandiose narcissistic tendencies demonstrate elevated confidence levels, a strong inclination toward achievement, and a readiness to assume leadership roles. These traits, particularly confidence and assertiveness, often facilitate their selection for managerial positions^6^. Kaiser^19^ outlined that an abundance of narcissistic traits is often perceived as highly detrimental within managerial positions. The literature highlights narcissistic leaders as penchant for exploiting others, making impulsive decisions, and engaging in unethical behavior adversely impacts relationships and team performance^20,21^. For instance, CEOs narcissism is positively related to the likelihood that an organization will be subjected to a lawsuit^22^, to the manipulation of the reported earnings^9^, to incurring significantly higher audit fees from external auditors^23^, and tending to be bold in their actions and often engage in substantial risk-taking so as to demonstrate their self-perceived superiority to others^24,25^. Moreover, research has identified a negative correlation between grandiose narcissism and crowdfunding success. Specifically, it suggests that the higher the level of narcissistic personality traits in an entrepreneur, the lower the likelihood of successfully funding their crowdfunding campaign^26^.

However, the literature also underscores that grandiose narcissism possesses a dual nature, with certain positive attributes. For instance, research indicates that narcissistic entrepreneurs tend to gravitate towards more innovative and risky venture opportunities^27^. Additionally, within entrepreneurial teams, higher levels of narcissism are linked to improved business planning performance^28^. Previous research indicates that narcissistic managers exhibit various favorable traits that yield tangible benefits for organizations. They often embody charismatic leadership qualities, effectively navigating organizations through crises^24,29,30^, strive for higher performance by stimulating innovation^31^, and fostering a heightened entrepreneurial spirit^32^. Recent studies, such as that by Böhm and Blickle^29^, suggest that narcissistic leaders, particularly those high in political skill, possess the self-discipline to regulate aggressive tendencies while skillfully presenting their desire for admiration, thereby garnering acceptance from subordinates.

Narcissistic CEOs often emerge as visionary leaders^30^, partly due to their consistently optimistic communication with stakeholders^33^. Their narcissism correlates positively with engagement in corporate social responsibility (CSR) initiatives^34,35^, likely driven by the desire to garner heightened admiration for both them and their organizations through increased CSR investment.

### Individual work performance

Work performance involves behaviors relevant to organizational goals and differs from productivity, which focuses on tangible outcomes. An employee’s effectiveness doesn’t always translate into high productivity due to various contextual factors. Evaluating individual performance encompasses goal-centric attitudes and actions like goal setting, time management, skill acquisition, and professional development. This multidimensional concept prioritizes observable behaviors over outcomes and spans task performance, contextual behaviors, and counterproductive work behaviors^36^.

Task performance refers to meeting job expectations in quantity, quality, essential skills, and professional knowledge. It includes planning, problem-solving, accuracy, knowledge maintenance, goal setting, and timely goal achievement. Contextual performance, also known as organizational citizenship behavior, goes beyond duties, encompassing actions like taking on extra tasks, initiating projects, engaging in collaborations, offering advice, and showing enthusiasm. However, counterproductive work behaviors (CWB) harm an organization, including complaints, negativity, off-task behavior, presentism, intentional mistakes, misuse of privileges, and exaggerating challenges.

Limited research has explored how narcissism influences individual work performance dimensions^37–39^. In managerial positions, the examination of narcissism primarily has focused on its correlation with task performance^40^. This study also considered other trait-based resources like effective coping strategies. Vulnerable narcissists struggle with self-worth, leading to sensitivity to criticism, social withdrawal, and engaging in CWB. Grandiose narcissists exhibit inflated self-importance, seeking admiration, and might display arrogant or exploitative behaviors^41^. Surprisingly, despite their traits, grandiose narcissists are less inclined to engage in CWB. Theoretically, shaping a distinct dynamic in their work performance, neurotic, vulnerable narcissists could potentially engage in CWB; however, this relationship has yet to be explored.

Narcissists generally tend to exaggerate their knowledge and possess an inflated self-centered view^42^. This would facilitate correlating positively with self-reported performance ratings among managers. However, challenges persist in task performance studies, prompting further investigation into social desirability responding in self-reported work performance.

### The role of core personality traits

One popular dimensional model of core personality traits is the Five Factor Model (FFM/Big Five)^43^, breaking down personality into five broad domains^44^: extraversion, neuroticism, agreeableness, conscientiousness, and openness. Another model is HEXACO model^45^, which in addition of the traits encompassing Big Five also reflects honesty-humility. The consideration of whether narcissism contributes to the prediction of individual work performance beyond the core personality traits holds significance for two main reasons. Firstly, the Big Five traits are widely acknowledged to cover a substantial portion of the personality domain, with several of these traits demonstrating predictive power for leadership^46^ and work-related ratings^47,48^. The trait most strongly predictive of job performance and a significant predictor of leadership, conscientiousness, generally shows little association with narcissism^49^.

Secondly, narcissism itself shares variability with some of the core traits, raising concerns about conceptual overlap. Specifically, grandiose narcissism exhibits positive correlations with extraversion and negative correlations with agreeableness and neuroticism^50^. In contrast, vulnerable narcissism is positively associated with neuroticism^14,50^. Both variants share aspects of exploiting others, relating negatively to honesty-humility^51^. Consequently, while controlling for the core traits may not diminish the impact of narcissism notably, it remains essential to include these traits particularly in work-related analyses for understanding the power of personality^52^.

### The current study

Our study’s first objective was to compare two variants of narcissism in managers and people from the general population. Theoretical underpinnings and empirical evidence, aligning the grandiose variant with extraversion and the vulnerable variant with neuroticism, were considered. Given the complexity of managerial roles, where grandiose traits tend toward dominance and vulnerability manifests as defensiveness and insecurity, the anticipation was that managers would exhibit higher levels of grandiose narcissism and lower levels of vulnerable narcissism compared to counterparts in the workplace and the general population^53^.

Our study’s second objective was, within the managerial sample, to investigate the divergent correlations between both forms of narcissism and core personality traits, alongside examining their correlations with socially desirable responding. Assuming universality of core personality traits in humans^54^, and considering personality theory and past research^15,38,39,50,55,56^, we expected grandiose narcissism to show a positive correlation with extraversion and a negative one with neuroticism and agreeableness, while vulnerable narcissism to be positively correlated with neuroticism and negatively with extraversion and agreeableness^57^. Previous studies^50,51,58^ have shown no notable link between conscientiousness and either form of narcissism. Therefore, we do not anticipate a significant relationship between conscientiousness and narcissism in our study. Moreover, we expected both grandiose narcissism^29,37,55,59^ and vulnerable narcissism^51^ to have negative correlations with honesty-humility. Narcissistic grandiosity may correlate with diminished levels of honesty-humility due to a proclivity for exploiting others. Conversely, narcissistic vulnerability is associated with abusive (aggressive) supervision tendencies^60^. Leaders harboring a vulnerable self-concept might resort to aggression against their followers, driven by internal attributions of failure and feelings of shame.

Our study’s third objective was to explore the associations between both variants of narcissism and managerial performance, encompassing task performance, contextual performance, and counterproductive work behavior. Different relationships with core personality traits would have implications for managers’ self-reports of their individual work performance. Acknowledging the limitations of self-reported work performance, our study aimed to control for core personality traits and socially desirable responses in these reports, ensuring a more comprehensive investigation. For instance, Ramos-Villagrasa et al.^39^ demonstrated that while grandiose narcissism moderately correlated with contextual performance, it showed no relation to task performance or CWB. Relationships between vulnerable narcissism and the three individual work performance dimensions among managers have not been previously explored.

We formulated the following hypotheses:

#### Hypothesis 1

Managers, due to their role in leading others, were expected to demonstrate elevated levels of grandiose narcissism and decreased levels of vulnerable narcissism compared to persons in non-managerial roles.

#### Hypothesis 2

- Grandiose narcissism would correlate positively with extraversion and negatively with agreeableness and neuroticism. It was also expected to exhibit a negative correlation with honesty-humility.
- Vulnerable narcissism would correlate positively with neuroticism and negatively with extraversion and agreeableness. Similar to grandiose narcissism, it was anticipated to exhibit a negative correlation with honesty-humility.
- It was not anticipated that conscientiousness would exhibit a significant correlation with either variant of narcissism.

#### Hypothesis 3

- Grandiose narcissism, linked positively with extraversion, was predicted to be associated with higher levels of contextual performance in managerial roles.
- Vulnerable narcissism, positively correlated with neuroticism, was expected to predict lower task performance and higher CWB among managers.

## Materials and methods

### Participants and procedure

The study involved 344 managers, 57.8% being women, employed in various sectors in Sweden. On average, they were 49 years old, with around five years of managerial experience in their current roles. The dataset comprised managers from nine distinct organizations, with 70% in human-oriented sectors and the remaining 30% in manufacturing industries with positions ranging from superior (19.4%), intermediate (68.6%), to lower levels like group leaders (12%). These managers worked across fields like industrial production, social services, nursing, care services, and education. They were employed by privately-owned companies (45%), or municipalities and state organizations (55%). Eight organizations were situated in western Sweden, with one privately-owned municipality situated in Stockholm.

The data for this study were gathered within a leadership-focused project led by the first author. They engaged Human Resources (HR) managers from both municipal and private sectors throughout western Sweden, extending invitations to managers within these organizations to partake in the study. The HR managers received comprehensive project information, including details about the questionnaires and their measurement criteria, alongside an ethics statement. Subsequently, they relayed this information to their organizations’ CEOs. Upon agreement to participate, the HR managers supplied mailing lists containing potential participants.

Managers were asked to complete a web-based questionnaire using Google Forms, a free Internet-based software. Given the anonymous nature of the survey, researchers were unaware of which managers had already responded. To ensure adequate participation, all managers on the mailing lists received three reminder emails. Data collection occurred over a five-week period.

The response rates from the participating organizations were satisfactory, averaging 73% with a range between 65 and 81%.

### Instruments

Aware of managers’ time constraints for lengthy surveys on psychological measures^61^, we opted for abbreviated versions of self-report instruments. A high scale score indicates a high value of the measured variable. We kept all items within the utilized instruments, even though this action may have slightly reduced the reliability measured by the scales’ Cronbach's alpha. Our goal was to enable a direct comparison of mean scale scores with other sample data. Notably, two variables (grandiose narcissism and openness) contained a few items that impacted their reliability. However, upon re-running the regression analyses, the results remained largely unchanged, showing only minor discrepancies.

#### Hypersensitive Narcissism Scale (HSNS)

The HSNS^57^ measures vulnerable narcissism using responses ranging 1–5, *Very uncharacteristic or untrue, strongly disagree* to *Very characteristic or true, strongly agree.* The index is derived from the sum of items, resulting in a possible range between 10 and 50. The Swedish version (translated by Björkman and Kajonius, revised by Hellström) was used (see Supplementary Information for HSNS in both English and Swedish).

#### Short dark triad (SD3)

The SD3^62^ comprises three scales, but only items from the subclinical Narcissism (the grandiose variant) scale were sampled. The SD3 uses responses ranging 1–5, from *Strongly disagree* to *Strongly agree*. The Swedish version (translated and adapted by Lindén and Dåderman) is published^63^.

#### Individual Work Performance Questionnaire (IWPQ)

The IWPQ^64^ comprises three scales: task performance, contextual performance, and counterproductive work behavior (CWB). IWPQ measures individual work performance using responses ranging 1–5, from *Seldom* to *Always* for task and contextual performance, and from *Never* to *Often* for CWB. All items have a recall period of 3 months. The Swedish version is published^7^.

#### Mini international personality item pool-6 inventory (Mini-IPIP6)

The Mini-IPIP6^65^ measures core personality traits comprising six scales: neuroticism, extraversion, openness, agreeableness, conscientiousness, and honesty-humility. The Mini-IPIP6 uses responses ranging 1–7, from *Strongly disagree* to *Strongly agree*. The Swedish version (translated and adapted by Backström, Dåderman, Grankvist, Kajonius, and Lundin) is published^63^.

#### Balanced inventory of desirable responding (BIDR 6)^66,67^

BIDR 6 comprises two measures for socially desirable responding using responses ranging 1–7, from *Not true at all* to *Completely true*. These can be separated into *unconscious* self-deceptive enhancement and *conscious* impression management^68,69^. Self-deceptive enhancement is a stable personality characteristic, while impression management depends on the characteristics of the situation a person is in^69^. The Swedish version (translated by Grankvist and Lundin) was used (see Supplementary Information for BIDR 6 in both English and Swedish).

### Data management and statistical analyses

All statistical analyses were performed in SPSS 28. Single missing values (< 1%) were replaced by the mean for all cases. We computed means and standard deviations of the variables. Internal consistency of the scales was determined using Cronbach’s alpha^70^. Because we used short scales comprising a few items, we also calculated mean inter-item correlations.

To evaluate Hypothesis 1, aimed at distinguishing between two distinct variants of narcissism among managers and the general population, we conducted one-sample *t*-tests across several samples. Data on narcissism were sourced from published studies or directly obtained from the authors of the Swedish versions of the SD3 and the HSNS. The weighted mean differences were calculated for the SD3 and HSNS narcissism average scores obtained by the authors of the citied studies (see Table 1 for details), and compared to our sample’s average score using one sample *t*-tests. In line with Erkoreka and Navarro^71^, it was imperative to recalculate the data collected from Hendin and Check’s^57^ samples.

**Table 1:** Narcissism variants comparing managers with seven samples from the general population.

Variant of narcissism	Managers *N* = 344 *M* (*SD*)	General population *M* (*SD*)	*N*, [*M* age years (*SD*); % males)]	*t*	*p*	*d*
Grandiose (SD3)	2.96 (0.49)	2.80 (0.88)^a^	387 [–; –]	5.86	< .001	0.22
		2.70 (0.60)^b^	228 [34.0 (12.6); 33.8]	9.61	< .001	0.47
		2.77 (0.61)^c^	853 [39.4 (11.8); 32.2]	6.98	< .001	0.34
		2.84 (0.69)^d^	324 [40.0 (11.3); 30.0]	4.36	< .001	0.20
Vulnerable (HSNS)	20.97 (5.12)	29.23 (5.67)^e^	403 [–; 35.5]	− 29.94	< .001	1.53
		25.75 (6.96)^f^	346 [42.0 (10.0); 14.5]	− 17.33	< .001	0.78
		30.49 (6.35)^g^	437 [23.0 (6.0); 38.2]	− 32.73	< .001	1.65

“–” undergrade students, *M* (*SD*) of age or % of males was not reported.

*SD3* Short Dark Triad, *HSNS* Hypersensitive Narcissism Scale. The effect sizes (Cohen’s *d*) were computed using an online tool, Effect Size Calculator (Cohen's D) for T-Test (socscistatistics.com). The weighted mean differences were calculated for the SD3 and HSNS narcissism average scores obtained by the following authors, and compared to our sample’s average score using one sample *t*-tests. Normative data on SD3 (undergraduate students) obtained from Paulhus.

^b–d^Data from working people in Sweden.

^b^published by Hjalmarsson and Dåderman^38^.

^c^submitted by Dåderman et al. (2024).

^d^published by Dåderman and Basinska^55^.

^e^Normative data (undergraduate students) on HSNS published by Hendin and Cheek^57^; *M* and *SD* recalculated.

^f^Data (*M* and *SD*) from the Swedish community sample obtained from Balder and Björkman, published by Kajonius and Björkman^16^.

^g^Data from the Polish community sample, published by Zajenkowski and Szymaniak^56^.

To evaluate Hypothesis 2, how the variants of narcissism relate to core personality traits, we employed Pearson correlation coefficients (see Table 2).

**Table 2:** Zero-order Pearson’s correlations between the study variables.

Variable	1	2	3	4	5	6	7	8	9	10	11	12	13	14
1. HSNS	(.73)													
2. SD3 N	**.02**	(.59)^a^												
3. IWPQ TP	− .26*	**.13**	(.72)											
4. IWPQ CP	− **.16**	.22*	.38*	(.81)										
5. IWPQ CWB	.47*	− **.01**	− .27*	− .21*	(.71)									
6. IPIP6 E	− .20*	.51*	.06	.28*	− .13	(.79)								
7. IPIP6 A	− .29*	**.06**	.00	.18*	− .20*	.27*	(.64)							
8. IPIP6 C	− .28*	**.08**	.36*	.18*	− .24*	.07	.08	(.73)						
9. IPIP6 N	.42*	**.00**	− .24*	− .17	.39*	− .09	− .04	− .22*	(.69)					
10. IPIP6 O	**.00**	**.12**	− .01	.18*	.00	.27*	.25*	− .10	− .00	(.57)^b^				
11. IPIP6 H–H	− .39*	− .33*	.03	.06	− .22*	.01	.29*	.03	− .27*	.08	(.64)			
12. BIDR 6 SDE	− **.10**	.23*	.23*	.17	− .11	.06	− .04	.31*	− .15	.03	− .13	(.70)		
13. BIDR 6 IM	− .44*	− **.05**	.19*	.11	− .40*	.05	.21*	.28*	− .29*	− .07	.31*	.21*	(.67)	
14. Sex	− .04	− .00	.01	.07	− .04	.04	.28*	.05	.02	.11	.11	− .06	.10	
15. Age	− .17	− .08	.13	.00	− .08	− .04	− .03	.04	− .14	− .06	.11	− .03	.10	− .05

*N* = 344, *HSNS* Hypersensitive Narcissism Scale, *SD3 N* Narcissism scale from the Short Dark Triad, *IWPQ* Individual Work Performance Questionnaire, *TP* Task Performance, *CP* Contextual Performance, *CWB* Counterproductive Work Behavior, *IPIP6* Mini International Personality Item Pool-6 Inventory, *E* Extraversion, *A* Agreeableness, *C* Consciousness, *N* Neuroticism, *O* Openness, *H–H* Honesty Humility, *BIDR 6* Balanced Inventory of Desirable Responding, *SDE* Self-Deceptive Enhancement, *IM* Impression Management.

^a^Excluding four items may heighten α to .62.

^b^Excluding one item may heighten α to .61. Sex: 1 = Male, 2 = Female. Correlations with the two variants of narcissism (*n* = 26) are Bonferroni adjusted; only correlations with p ≤ .0019 reach significance at *p* < .05 (.05/26 = .0019) after adjustment. Bonferroni adjusted correlations and those < .001 are marked med *. The bold values represent the correlations between two different forms of narcissism and the specific variables that are the main focus of investigation within the study. Cronbach’s alpha (α) values are on the diagonal. Mean inter-item correlations were all within acceptable values (.20–.40).

To evaluate Hypothesis 3, how these predict managerial work-related performance, we performed three hierarchical regression analyses. Both variants of narcissism were entered as predictors in the first step. Subsequently, all six core personality traits were included in the second step, followed by the two scales measuring socially desirable responding in the final step.

We adhered to effect size guidelines in individual differences research, such as considering *r* = 0.20–0.30 as indicative of a medium effect^72^. Cohen’s *d* values are typically interpreted as follows: 0.20 represents a small effect (might not be discernible to the naked eye), 0.50 signifies a medium effect, and 0.80 indicates a large effect (are easily visible without aid)^73^.

### Ethical statement

This study adhered to the Swedish Research Council’s guidelines. Data were collected in 2017 in accordance with Swedish law (2003:460, §2). Approval was secured from participating organization leaders, and the project’s data handling was officially sanctioned by Municipal Academy West (Diary no. 100127). All experimental protocols were approved by the Ethics Committee at Lund University. Prior to accessing the questionnaire, participants were informed about purpose of this study, and that their participation was voluntary and confidential, with guaranteed anonymity and the option to withdraw at any time. The questionnaire did not inquire about sensitive personal data. Written informed consent was obtained from all participants following the Declaration of Helsinki.

## Results

### How does narcissism vary between managers and people from the general population?

Table 1 demonstrates a notable distinction between the present group of managers and samples from the general population. Particularly, these managers showcased significantly higher mean scores in measuring grandiose narcissism and notably lower scores in vulnerable narcissism. Therefore, Hypothesis 1 finds support. The observed variance ranged from small to considerable for grandiose narcissism and notably substantial for vulnerable narcissism.

### The role of core personality traits

Table 2 illustrates the correlations between core personality traits, socially desirable responding, and the two observed variants of narcissism within our manager sample. In line with Hypothesis 2a, grandiose narcissism showed a strong positive correlation with extraversion. However, contrary to Hypothesis 2a, grandiose narcissism did not display the expected negative correlations with neuroticism and agreeableness. Similarly, in line with Hypothesis 2b, vulnerable narcissism showed strong positive correlations with neuroticism and negative correlations with extraversion and agreeableness. Both narcissism variants demonstrated clear negative correlations with honesty-humility, aligning with Hypotheses 2a and 2b. Contrary to Hypothesis 2c, which posited that conscientiousness would not significantly correlate with either variant of narcissism, it is notable that grandiose narcissism unexpectedly displayed a negative significant correlation with conscientiousness. However, in line with Hypothesis 2c, vulnerable narcissism did not exhibit a significant correlation with conscientiousness. Consistent with Hypotheses 3a and 3b, the correlations between the two narcissism variants and the three dimensions of individual work performance revealed contrasting trends, prompting further investigation through regression analyses in subsequent steps.

We incorporated measures to account for socially desirable responses. Notably, impression management, reflecting conscious misrepresentation, strongly correlated negatively with vulnerable narcissism, CWB, and neuroticism, while positively correlating with honesty-humility. Meanwhile, self-deceptive enhancement, a subconscious positivity bias in responses, displayed a strong positive correlation solely with conscientiousness.

### Predictions of managerial work performance

In order to test hypothesis 3, we performed regression analyses on work performance (IWPQ), using the dimensions of task performance, contextual performance, and CWB as dependent variables. To isolate the distinct impact of narcissism while accounting for core personality traits and socially desirable responding, we conducted three separate hierarchical linear regressions, one for each dependent variable. The detailed outcomes of these analyses can be found in Table 3.

**Table 3:** Hierarchical regression analyses of three dimensions of work performance (IWPQ).

Explained variances per step	Task performance			Contextual performance			CWB		
	*R*	Δ*R*^2^	*p*	*R*	Δ*R*^2^	*p*	*R*	Δ*R*^2^	*p*
Step 1	.29	**.08**	< .001	.27	**.07**	< .001	.47	**.22**	< .001
Step 2	.44	**.11**	< .001	.39	**.08**	< .001	.53	**.06**	< .001
Step 3	.46	.01	.206	.40	.01	.260	.56	**.03**	< .001

Coefficients Step 3	β	*p*		β	*p*		β	*p*	
HSNS (vulnerable)	− **.15**	.015		− .01	.913		**.26**	< .001	
SD3 N (grandiose)	.11	.075		.10	.110		− .01	.909	
IPIP6 Extraversion	− .06	.346		**.15**	.017		− .02	.715	
IPIP6 Agreeableness	− .07	.235		.08	.171		− .07	.195	
IPIP6 Consciousness	**.27**	< .001		.11	.055		− .06	.220	
IPIP6 Neuroticism	− **.11**	.047		− .10	.083		**.21**	< .001	
IPIP6 Openness	− .01	.819		**.12**	.034		.002	.961	
IPIP6 Honesty-Hum	.04	.441		.04	.540		.02	.726	
BIDR 6 SDE	.09	.110		.09	.114		.01	.832	
BIDR 6 IM	.03	.626		.01	.877		− **.20**	< .001	

See Table 2 for abbreviations of the instruments. Δ*R*^2^ = coefficient of determination change. Step 1: HSNS (measures vulnerable narcissism) and SD3 N (measures grandiose narcissism). Step 2: all six core personality traits. Step 3: the two scales measuring socially desirable responding. Bold values correspond to statistically significant associations, *p* < .05.

In summary, after controlling for the role of core personality traits and socially desirable responding, grandiose narcissism didn’t predict work performance variables, while vulnerable narcissism and neuroticism predicted higher CWB and lower task performance. Conscientiousness stood out as the most influential predictor of task performance, while extraversion as the most influential predictor of contextual performance. These findings were adjusted for self-deceptive enhancement, a variable found to have no significant influence on any dimension of individual work performance. Impression management, however, negatively influenced CWB.

## Discussion

This study aimed to bridge existing gaps in managerial literature by shedding light on the comparison of mean values of narcissistic variants among managers with people from the general population. It examined their divergent correlations with core personality traits, while also considering socially desirable responding. Furthermore, it explored their associations with various forms of managerial performance, encompassing task performance, contextual performance and counterproductive work behavior.

Only a few studies^37–39^ have investigated narcissism’s relationships with the three dimensions of individual work performance^36^. However, these studies encompassed broader participant groups beyond managerial roles and didn’t specifically target vulnerable narcissism. Among these, Ramos-Villagrasa et al.^39^ revealed a noteworthy correlation (*r* = 0.23) between task performance and grandiose narcissism. Interestingly, existing findings consistently showed a moderate correlation (approximately *r* ~ 0.20) between grandiose narcissism and contextual performance, including our study. However, after controlling for core personality traits and social desirability responding, this significant association disappeared (Table 3), which other studies have not analyzed. Notably, our study unveiled consciousness as the most influential predictor of contextual performance, a novel but not surprising discovery. Aligning with prior research, our study corroborated the minimal impact of grandiose narcissism on CWB.

Our study expanded upon prior research, particularly Miller et al.^50^, by examining a sample comprised exclusively of managers, revealing an absence of significant correlation between grandiose leadership and vulnerable narcissism (Table 2). The table highlights a novel finding within the managerial sample: contrary to Hypothesis 2a, grandiose narcissism did not exhibit the anticipated negative correlations with neuroticism and agreeableness. Additionally, it is noteworthy that grandiose narcissism unexpectedly demonstrated a negative significant correlation with conscientiousness. These results diverge from those observed in the general population ^50,56^. We will endeavor to elucidate these inconsistencies. These findings may be illuminated by the distinct differences in narcissistic tendencies observed between our manager-only sample and samples drawn from the general population. Specifically, managers exhibited higher levels of grandiose narcissism and lower levels of vulnerable narcissism (see Table 1). Another potential explanation for the lack of a negative correlation between grandiose narcissism in managers and agreeableness could be attributed to a prevalent cultural norm in Swedish workplaces known as “jäntelagen,” which emphasizes a tendency to agree and maintain politeness with coworkers. Agreeableness reflects a disposition towards trust, compassion, and kindness. Managers high in agreeableness tend to foster positive interpersonal connections, prioritize cooperation, and seek to prevent conflicts. The majority of the participants are women, and it is well-documented that women typically exhibit higher levels of agreeableness compared to men^44^. Likewise, the absence of a negative correlation between high grandiose narcissism and neuroticism may be attributed to a cultural norm prevalent in Swedish workplaces, characterized by strong employment regulations that ensure job security.

A final notable contribution of our study was the exploration of vulnerable narcissism’s impact on the three dimensions of individual work performance (see Table 3). Through regression analyses, while adjusting for core personality traits and socially desirable responding, our results indicated that only vulnerable narcissism and neuroticism emerged as significant predictors of CWB. Vulnerable narcissism also negatively predicted task performance, although the strength of this association was limited. Furthermore, our study unveiled that grandiose narcissism related positively to self-deceptive enhancement, while vulnerable narcissism related negatively with impression management—a novel finding likely unreported in prior literature.

### Strengths and limitations

While the study’s cross-sectional design presents a limitation, it is noteworthy that the current research achieved a commendably high response rate (73%), which enhances the representativeness of the conclusions drawn for Swedish managers. A notable strength lies in the sample size, which encompasses leaders across diverse managerial roles and organizations. This diversity in the sample enhances validity by introducing greater variation, enabling robust analyses. Importantly, our findings suggest a potential universal efficacy of specific managerial qualities across varied organizational settings. This strength contrasts with past research, as noted by Cycyota and Harrison^74^, where obtaining high response rates and large sample sizes was challenging and often resulted in limited data availability.

Another notable aspect of this leadership project lies in the thoroughness of the managers’ participation, as evidenced by their completion of the comprehensive survey encompassing various psychological measures^40^. Bednar and Westpha ^61^ observed that managers, particularly those in senior positions, seldom recognize the value or find the time to undertake lengthy surveys pertaining to psychological measures. While these measures have been translated and justified for use^40,52,75^, understanding any nuances regarding how narcissism presents in Sweden compared to other contexts would be beneficial. Consistent and reliable results across Western countries have, however, not been found. Nevertheless, utilizing different instruments than those used in our study, there seems to be a discernible trend indicating that in more modern, progressive, and individualistic societies, there is a lower prevalence of narcissism^76,77^.

Moreover, the study’s strength also lies in its approach to evaluating work performance, encompassing not just task-oriented metrics but also various other performance indicators. This inclusive evaluation acknowledges the nuanced nature of managerial behavior, emphasizing the need for a balanced focus on both task-oriented (such as transactional behaviors, initiating structure, and boundary spanning) and person-oriented (such as transformational behaviors, consideration, empowerment, and motivational behaviors) leadership styles^78^.

Although gathering self-report data can pose challenges, it is crucial to acknowledge the potential biases inherent in such data, which could have impacted the precision of trait measurements. This concern is salient when evaluating traits such as grandiose narcissism, often viewed as advantageous in leaders. Despite attempts to alleviate social desirability bias, inherent limitations endured. Self-reported evaluations, susceptible to social desirability bias, are prone to distortion, especially among persons with narcissistic tendencies. This distortion often manifests as exaggerated or fluctuating self-appraisals. Narcissists are known for their adeptness at deceptive communication^79^ with a strong inclination towards self-enhancement and perceived superiority^80,81^, particularly concerning positive traits and their perceived leadership competence. Table 3 indicates some of these tendencies. Notably, the positive association between impression management—conscious attempts to manipulate perceptions—and honesty-humility aligns with the societal preference for honest leaders. Conversely, impression management exhibited negative correlations with CWB and neuroticism, both widely recognized as undesirable traits in managerial roles. This suggests a deliberate and subconscious drive among managers to present themselves as task-oriented leaders, both for their self-image and others’ perceptions.

### Conclusions and implications

This study serves as a foundational step towards empirical comprehension, seamlessly integrating with existing literature. One conclusion is that vulnerable narcissism (and neuroticism) exhibited a detrimental impact on task performance, while grandiose narcissism (and extroversion) had a positive relationship. Neither form of narcissism was significantly related to contextual performance, while counterproductive work behavior was clearly associated with vulnerable narcissism (and neuroticism). Our conclusions suggest that organizations emphasizing task performance in managers may not benefit from managers exhibiting vulnerable narcissism or high neuroticism. Instead, recruiting managers with high extroversion and conscientiousness could be more advantageous.

### Supplementary Information

Supplementary Information.

## Data Availability

The data underlying the results presented in the study are published 27 December 2023, and available on Mendeley Data (10.17632/94rsp7bw9x.1).
