# Supplementary material for: Linking grandiose and vulnerable narcissism to managerial work performance, through the lens of core personality traits and social desirability
Source: Sci Rep. 2024 May 28;14:12213. doi: 10.1038/s41598-024-60202-7 (PMC11133368; doi:10.1038/s41598-024-60202-7)
Supplement: Supplementary file 1 — Supplementary Information. [file 41598_2024_60202_MOESM1_ESM.docx]

**Supplementary Information**

**Article:** “Linking Grandiose and Vulnerable Narcissism to Managerial Work Performance, Through the Lens of Core Personality Traits and Social Desirability”

**Authors:** Anna M. Dåderman and Petri J. Kajonius

**Journal:** *Scientific Reports*

This Supplementary Information contains Swedish translations of two scales used in the current study, the Hypersensitive Narcissism Scale (HSNS; Hendin & Cheek, 1997), and the Balanced Inventory of Desirable Responding (BIDR 6; Bobbio & Manganelli, 2011; Paulhus, 1984, 1991).

**The Swedish Version of the Hypersensitive Narcissism Scale**, translated 2016 by Björkman and Kajonius, revised 2017 by Hellström.

Instruction [Instruktion]: Questions about your characteristic patterns of feeling, thinking and behaving. Mark how much each statement applies to you. [Frågor om dina karakteristiska mönster av känslor, tänkande och beteende.  Markera hur mycket varje påstående stämmer in på dig.]

Answer options [Svarsalternativ]:

1 = Very uncharacteristic or untrue; strongly disagree [Stämmer inte alls; så gott som aldrig]

2 = Does not fit well; not often [Stämmer inte bra; inte ofta]

3 = Sometimes true; sometimes not [Stämmer ibland; ibland inte]

4 = Fits well; often [Stämmer bra; ofta]

5 = Very characteristic or true; strongly agree [Stämmer mycket väl; så gott som alltid]

1. I can become entirely absorbed in thinking about my personal affairs, my health, my cares, or my relations to others. [Jag kan gå helt upp i mina personliga angelägenheter, min hälsa, mina omsorger eller mina relationer till andra.]

2. My feelings are easily hurt by ridicule or by the slighting remarks of others. [Mina känslor blir lätt sårade av andras förlöjligande eller nedlåtande anmärkningar.]

3. When I enter a room I often become self-conscious and feel that the eyes of others are upon me. [När jag går in i ett rum blir jag ofta generad och känner andras ögon på mig.]

4. I dislike sharing the credit of an achievement with others. [Jag ogillar att dela äran för en prestation med andra.]

5. I dislike being with a group unless I know that I am appreciated by at least one of those present. [Jag ogillar att vara i en grupp om jag inte vet att minst en i gruppen uppskattar mig.]

6. I feel that I am temperamentally different from most people. [Jag känner att jag har ett annat temperament än de flesta andra.]

7. I often interpret the remarks of others in a personal way. [Jag tar ofta andras anmärkningar personligt.]

8. I easily become wrapped up in my own interests and forget the existence of others. [Jag fastnar lätt i mina egna intressen och glömmer helt bort att det finns andra människor.]

9. I feel that I have enough on my hands without worrying about other people’s troubles. [Jag känner att jag har tillräckligt att sköta om, utan att oroa mig för andras problem.]

10. I am secretely “put out” when other people come to me with their troubles, asking me for my time and sympathy. [Jag blir i hemlighet irriterad när människor kommer till mig med sina problem och vill ha min tid och min sympati.]

**The Swedish Version of the Balanced Inventory of Desirable Responding** (BIDR 6; Bobbio & Manganelli, 2011, Appendix; Paulhus, 1984, 1991), translated 2016 by Grankvist and Lundin**.** The permission to translate and adopt to Swedish, as well as use it was given by Paulhus to the first author (AMD). Self-deceptive enhancement is evaluated by items 1-8, and impression management by items 9-16. (R) = reversed score.

Instruction [Instruktion]: Using the scale below as a guide, write a number next to each statement to indicate the degree to which it is true. [Använd skalan nedan som guide, skriv en siffra bredvid varje påstående för att ange i vilket grad det är sant.]

+ + + + + + +

1 2 3 4 5 6 7

Not true at all Partly true Completely true

[Inte alls sant] [Delvis sant] [Helt och hållet sant]

_____ 1. My first impressions of people usually turn out to be right. [Mitt första intryck av människor visar sig vanligtvis vara korrekt.]

_____ 2. I always know why I like things. [Jag vet alltid varför jag gillar saker och ting.]

_____ 3. Once I have made up my mind, other people can seldom change my opinion. [När jag väl bestämt mig så kan andra människor sällan få mig att ändra åsikt.]

_____ 4. I am fully in control of my own fate. [Jag har full kontroll över mitt framtida liv.]

_____ 5. I never regret my decisions. [Jag ångrar aldrig de beslut som jag har tagit.]

_____ 6. I am a completely rational person. [Jag är en fullständigt rationell person.]

_____ 7. I am very confident in my judgements. [Jag är väldigt säker på att mina omdömen är korrekta.]

_____ 8. It is all right with me if some people happen to dislike me. [För min del är det helt okej om en del människor inte tycker om mig.]

_____ 9. I sometimes tell lies, if I have to. [Om jag måste så ljuger jag ibland.] (R)

_____ 10. There have been occasions when I have taken advantage of someone. [Det finns tillfällen då jag utnyttjat någon annan.] (R)

_____ 11. I always obey laws, even if I am unlikely to get caught. [Jag gör alltid som man ska enligt lagen, även om det är osannolikt att jag skulle åka fast.]

_____ 12. I have said something bad about a friend behind his or her back. [Jag har sagt dåliga saker om en vän bakom ryggen på honom or henne.] (R)

_____ 13. I never try to hide my mistakes. [Jag försöker aldrig dölja mina misstag.]

_____ 14. I have done things that I do not tell other people about. [Jag har gjort sådant som jag inte berättar om för andra.] (R)

_____ 15. I have taken sick-leave from work or school even though I was not really sick. [Jag har sjukanmält mig från arbetet eller skolan trots att jag egentligen inte var sjuk.] (R)

_____ 16. I have some pretty awful habits. [Jag har en del ganska förfärliga vanor.] (R)

**References**

Bobbio, A., & Manganelli, A. M. (2011). Measuring social desirability responding. A short version of Paulhus’ BIDR 6. *TPM, 18,* 117-135.

Hendin, H. M., & Cheek, J. M. (1997). Assessing hypersensitive narcissism: A re-examination of Murray’s Narcissism scale. *Journal of Research in Personality, 31,* 588–599.

Paulhus, D. L. (1984). Two-component models of socially desirable responding. *Journal of Personality and Social Psychology, 46*, 598–609.

Paulhus, D. L. (1991). Measurement and control of response bias. In J. P. Robinson, P. R. Shaver, & L. S. Wrightsman (Eds.), *Measures of personality and social psychological attitudes* (pp.17-59). San Diego: Academic Press.
